# Epidemiological and clinical characteristics of hospitalized patients with cardiomyopathies in China: a nationwide retrospective analysis

**DOI:** 10.1186/s12889-026-28501-y

**Published:** 2026-07-10

**Authors:** Xinglin Yang, Ye Jin, Xudong Ma, Kai Sun, Ying Shi, Lanxia Gan, Jingya Zhou, Cheng Pang, Yi Wang, Limeng Chen, Haibo Wang, Zhuang Tian, Shuyang Zhang

**Affiliations:** 1 National Center for Healthcare Quality Management in Rare Diseases, Peking Union Medical College Hospital, Chinese Academy of Medical Sciences and Peking Union Medical College, Beijing, People’s Republic of China; 2 Department of Cardiology, Peking Union Medical College Hospital, Chinese Academy of Medical Sciences and Peking Union Medical College, No.1 Shuaifuyuan, Dongcheng District, Beijing, 100730 People’s Republic of China; 3 Center for Prevention and Early Intervention, National Infrastructures for Translational Medicine, Institute of Clinical Medicine, Peking Union Medical College Hospital, Chinese Academy of Medical Science and Peking Union Medical College, Beijing, People’s Republic of China; 4 Department of Medical Administration, National Health Commission of the People’s Republic of China, Beijing, People’s Republic of China; 5 Research Centre of Big Data and Artificial Intelligence for Medicine, The First Affiliated Hospital of Sun Yat-Sen University, Building A, Guangzhou Life Science Innovation Center, No. 51 Spiral Avenue, Haizhu District, Guangzhou, Guangdong 510700 People’s Republic of China; 6 Department of Medical Records, Peking Union Medical College Hospital, Chinese Academy of Medical Sciences and Peking Union Medical College, Beijing, People’s Republic of China; 7 Department of Nephrology, Peking Union Medical College Hospital, Chinese Academy of Medical Sciences and Peking Union Medical College, Beijing, People’s Republic of China; 8 Department of International Medical Service, Peking Union Medical College Hospital, Chinese Academy of Medical Sciences and Peking Union Medical College, Beijing, People’s Republic of China

**Keywords:** Health services accessibility, Incidence, Inpatients, Rare diseases, Resource allocation

## Abstract

**Background:**

Cardiomyopathies are major causes of heart failure and sudden cardiac death, but nationwide epidemiological data from China remain limited. Following the inclusion of several cardiomyopathies in the first national rare disease list in 2018, contemporary trends in hospitalization and in-hospital outcomes have not been systematically evaluated. This study aimed to characterize national hospitalization patterns for cardiomyopathies from 2018 to 2022.

**Methods:**

We conducted a nationwide retrospective study using the Hospital Quality Monitoring System (HQMS), which covers secondary and tertiary hospitals across 31 provinces. Cardiomyopathies were identified using National Clinical Version 2.0 codes. We assessed age- and sex-standardized hospitalization rates, regional variation, comorbidities, device use, intensive care unit (ICU) admission, in-hospital mortality, length of stay, and hospital costs. Temporal trends were analyzed using Joinpoint regression to estimate the average annual percent change (AAPC).

**Results:**

A total of 1,935,421 hospitalizations from 943,178 unique patients were included. The standardized hospitalization rate increased from 151.55 to 206.23 per 1,000,000 population (AAPC = 7.68%). Dilated cardiomyopathy was the most frequently recorded subtype. Men accounted for 67.99% of patients and were diagnosed at a younger age than women. Considerable provincial variation was observed. ICU admission increased from 2.49% to 6.97% (AAPC = 33.94%, *p* = 0.01), while in-hospital mortality remained stable (0.85% to 0.89%; AAPC = − 0.03%, *p* = 0.96). Median length of stay decreased from 8 to 7 days (AAPC = − 2.91%, *p* = 0.03), and median hospital costs increased from CNY 7,362 to 7,985 (AAPC = 2.13%, *p* = 0.03).

**Conclusions:**

Cardiomyopathy hospitalizations in China increased markedly between 2018 and 2022, with notable sex, age, and regional disparities. Rising ICU utilization alongside stable mortality suggests evolving case mix and improvements in acute care. These nationwide findings highlight unmet diagnostic and healthcare needs.

**Trial registration:**

Clinical trial number: not applicable.

**Supplementary Information:**

The online version contains supplementary material available at https://doi.org/10.1186/s12889-026-28501-y.

## Introduction

Cardiomyopathies are a diverse group of disorders characterized by structural and functional abnormalities of the cardiac muscle, distinct from more prevalent cardiovascular conditions [1]. Their primary forms include dilated (DCM), hypertrophic (HCM), restrictive (RCM), arrhythmogenic right ventricular cardiomyopathy (ARVC), and left ventricular noncompaction (LVNC) [1]. With a wide range of clinical manifestations and strong genetic predispositions, these disorders significantly contribute to heart failure and sudden cardiac death, posing a substantial public health burden [2–5].

Despite this burden, contemporary nationwide epidemiological data on cardiomyopathies in China are scarce. Most existing studies have been confined to selected regions, single centers or specific cardiomyopathy subtypes, leaving major gaps in our understanding of national trends in hospitalization, clinical characteristics and healthcare utilization. This evidence gap hampers effective service planning, resource allocation and the development of targeted strategies for earlier diagnosis and specialized care.

In 2018, the inclusion of primary cardiomyopathies in China’s First Rare Disease List marked a pivotal policy milestone in their recognition and management [6]. The List, jointly issued in May 2018 by the National Health Commission and four other central ministries, comprises 121 rare diseases and serves as the foundational national document for rare disease policy, drug approval, and reimbursement in China. Primary cardiomyopathies are included under the ‘idiopathic cardiomyopathy’ entry of the List, separate from secondary cardiomyopathies of identifiable upstream cause. Since then, initiatives such as genetic testing programs, multidisciplinary consultation platforms and rare disease registries have been introduced to enhance diagnosis, standardize treatment and promote more equitable access to care. However, the impact of these efforts on the epidemiology of cardiomyopathies and on real-world hospital care patterns has not been comprehensively evaluated at a national level.

Therefore, this study aimed to address these gaps by analyzing the epidemiological and clinical characteristics of patients hospitalized with cardiomyopathies in China from 2018 to 2022. Using a large-scale national inpatient database, we examined trends in hospitalization, patient demographics, comorbidities, clinical management and resource utilization, with the goal of providing evidence to inform future policy and health-system planning during this period of heightened rare-disease policy attention.

## Methods

### Study design

This nationwide retrospective study was conducted using data from the National Health Commission’s Hospital Quality Monitoring System (HQMS). The study was conducted and reported in accordance with the Strengthening the Reporting of Observational Studies in Epidemiology (STROBE) guidelines. The study protocol adhered to the ethical principles of the Declaration of Helsinki and was reviewed and approved by the Ethics Committee of Peking Union Medical College Hospital. The requirement for informed consent was waived because of the de-identified nature of the data.

### Study population

This nationwide retrospective study analyzed hospitalization records retrieved from the HQMS database across 31 provinces in China between January 1, 2018, and December 31, 2022. A total of 1,935,421 hospital admissions were included in the final analysis.

Patients were identified using the National Clinical Version 2.0 (NCV 2.0) codes for the five primary types of cardiomyopathies (DCM, HCM, RCM, ARVC, and LVNC), as detailed in Table S1. The NCV 2.0 is an adaptation of ICD-10 used for diagnostic coding in China. These diagnostic codes were extracted from either the principal or any secondary position on the discharge record. This pre-specified, all-position strategy was employed because acute manifestations precipitating admission (e.g., decompensated heart failure, arrhythmias) frequently occupy the principal diagnostic field under administrative coding conventions, while the underlying cardiomyopathy is recorded secondarily. Individuals assigned the unspecified-cardiomyopathy code (I42.900x003)—indicating a documented cardiomyopathy without morphological specification—were retained in the All-CM cohort for disease-burden estimation but omitted from subtype-specific analyses; this code is independent of the DCM (I42.0) cohort and the other four phenotypic subtypes. Records were excluded if they had missing patient identifiers, unspecified sex, or invalid data for age, length of hospital stay, or hospitalization costs. The reliability of HQMS data for nationwide epidemiological research has been established in previous studies [7, 8], which operated under identical data governance and privacy regulations.

### Statistical analysis

All analyses in this study were performed on a pre-specified patient-year analytic dataset. To avoid overweighting patients with frequent within-year readmissions, the raw extraction of admission records was deduplicated to one record per patient per calendar year, retaining each patient’s first admission of the year. This patient-year unit was applied uniformly across all reported analyses in this study for internal methodological consistency. Annual age- and sex-standardized hospitalization rates for cardiomyopathies from 2018 to 2022 were calculated. These rates were standardized to the 2020 Chinese standard population, derived from the Seventh National Population Census data obtained from the National Bureau of Statistics of China (http://www.stats.gov.cn). The 95% confidence intervals for the standardized rates were computed using Poisson distributions. In addition, cardiomyopathy hospitalizations were standardized against annual national hospital admission totals, obtained from the National Health Commission of China’s Statistical Bulletin on the Development of Health and Wellness in China, and expressed per 100,000 national hospital admissions to account for pandemic-era variation in overall hospital utilization. Descriptive statistics were employed to summarize the demographic and clinical characteristics of the patients. The normality of continuous variables was assessed using graphical methods (histograms and Q-Q plots) and descriptive statistics (skewness and kurtosis). Variables approximating a normal distribution (e.g., age at diagnosis) were expressed as mean ± standard deviation. Variables with skewed distributions (e.g., length of hospital stay, hospitalization costs) were expressed as median and interquartile range (IQR). Categorical variables were expressed as frequencies and percentages. Temporal trends were analyzed using the Joinpoint Regression Program version 5.1.0.0 (National Cancer Institute, Rockville, Maryland, USA), applying the grid-search method on log-linear models with the maximum number of joinpoints set to 1. Model selection was performed using the Bayesian Information Criterion (BIC). Annual percent change (APC) was computed for each identified segment and average annual percent change (AAPC) was computed across the entire study period (2018–2022), with 95% confidence intervals derived from the Empirical Quantile method. Other statistical analyses were performed using Python (version 3.12.3; Python Software Foundation, Wilmington, Delaware, USA) and R software (version 4.2.2; R Foundation for Statistical Computing, Vienna, Austria). Choropleth maps displaying geographic variations in hospitalization rates across China were generated using QGIS (version 3.36.1; QGIS Development Team, Open-Source Geospatial Foundation). For all analyses, a two-tailed *p*-value < 0.05 was considered statistically significant.

## Results

### Annual inpatient cases of cardiomyopathies

**Fig. 1:**
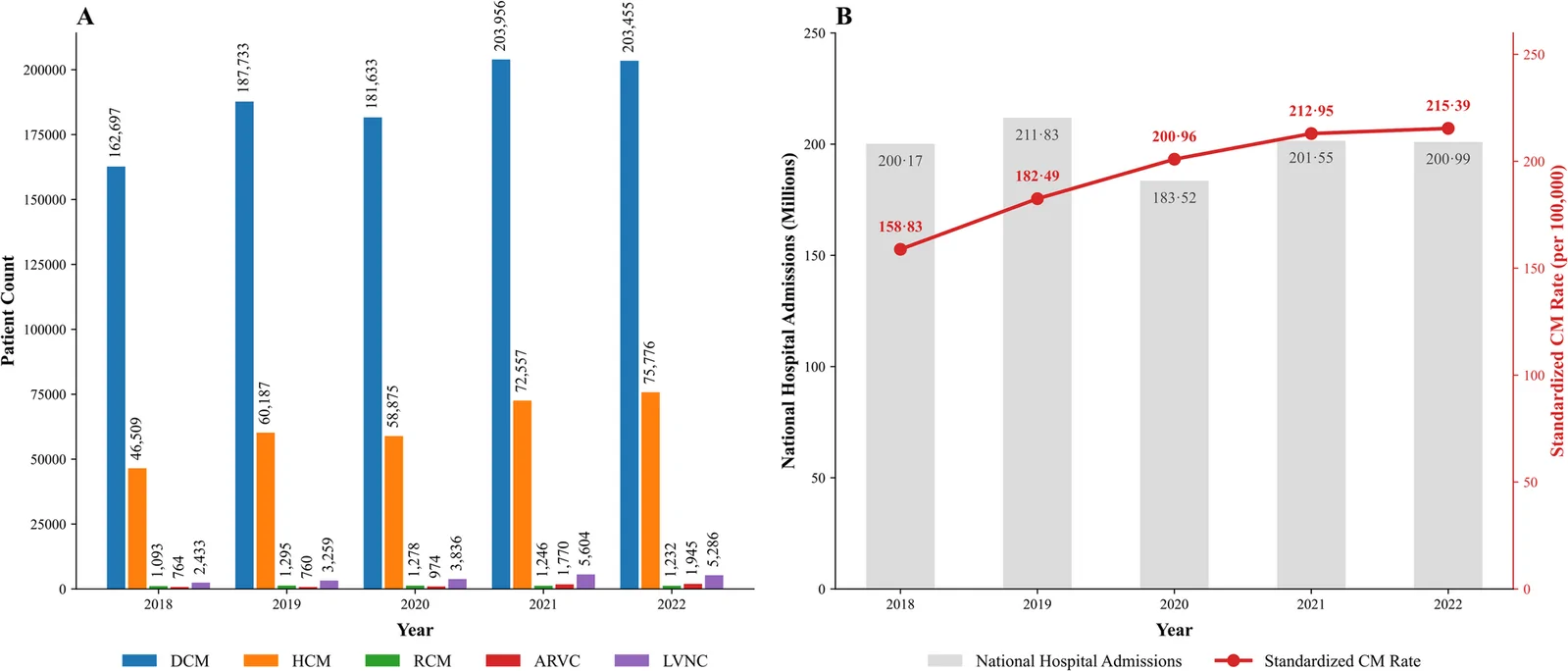
Annual cardiomyopathy hospitalizations in China, 2018–2022. **A** Year-deduplicated patient counts by subtype. The annual sum of the five subtypes corresponds to 98.7–99.4% of the All-CM cohort; the remainder represents patients with cardiomyopathy codes not assigned to one of the five primary phenotypic subtypes per the pre-specified analytic protocol. **B** Standardized cardiomyopathy hospitalization rate per 100,000 national hospital admissions (red line, right axis); national hospital admissions in millions (grey bars, left axis), from the National Health Commission’s annual Statistical Bulletin on the Development of Health and Wellness in China. DCM, dilated cardiomyopathy; HCM, hypertrophic cardiomyopathy; RCM, restrictive cardiomyopathy; ARVC, arrhythmogenic right ventricular cardiomyopathy; LVNC, left ventricular noncompaction; CM, cardiomyopathy

Between 2018 and 2022, an initial extraction yielded 2,087,157 cardiomyopathy-related hospital admission records. After data cleaning (Figure S1), 1,935,421 admissions met the inclusion criteria, corresponding to 943,178 unique patients across the 5-year period. Under the pre-specified patient-year analytic protocol (Methods), records were deduplicated to one per patient per calendar year, yielding 214,274 patients in 2018, 254,526 in 2019, 248,421 in 2020, 288,102 in 2021, and 291,464 in 2022 (Fig. 1A; total 1,296,787 patient-years). The five primary cardiomyopathy subtypes accounted for 98.7–99.4% of these patient-years; the remaining 0.4–1.3% comprised patients with cardiomyopathy diagnosis codes that did not map to one of the five primary phenotypic subtypes, retained in the All-CM cohort but not analyzed as separate sub-cohorts.

Notable variations were observed in the distribution of hospitalized patients across the five cardiomyopathy types (Fig. 1A). DCM accounted for the largest proportion of hospitalizations, followed by HCM. In contrast, RCM, ARVC, and LVNC accounted for much smaller proportions.

### Sex and age at diagnosis of patients with cardiomyopathies

**Fig. 2:**
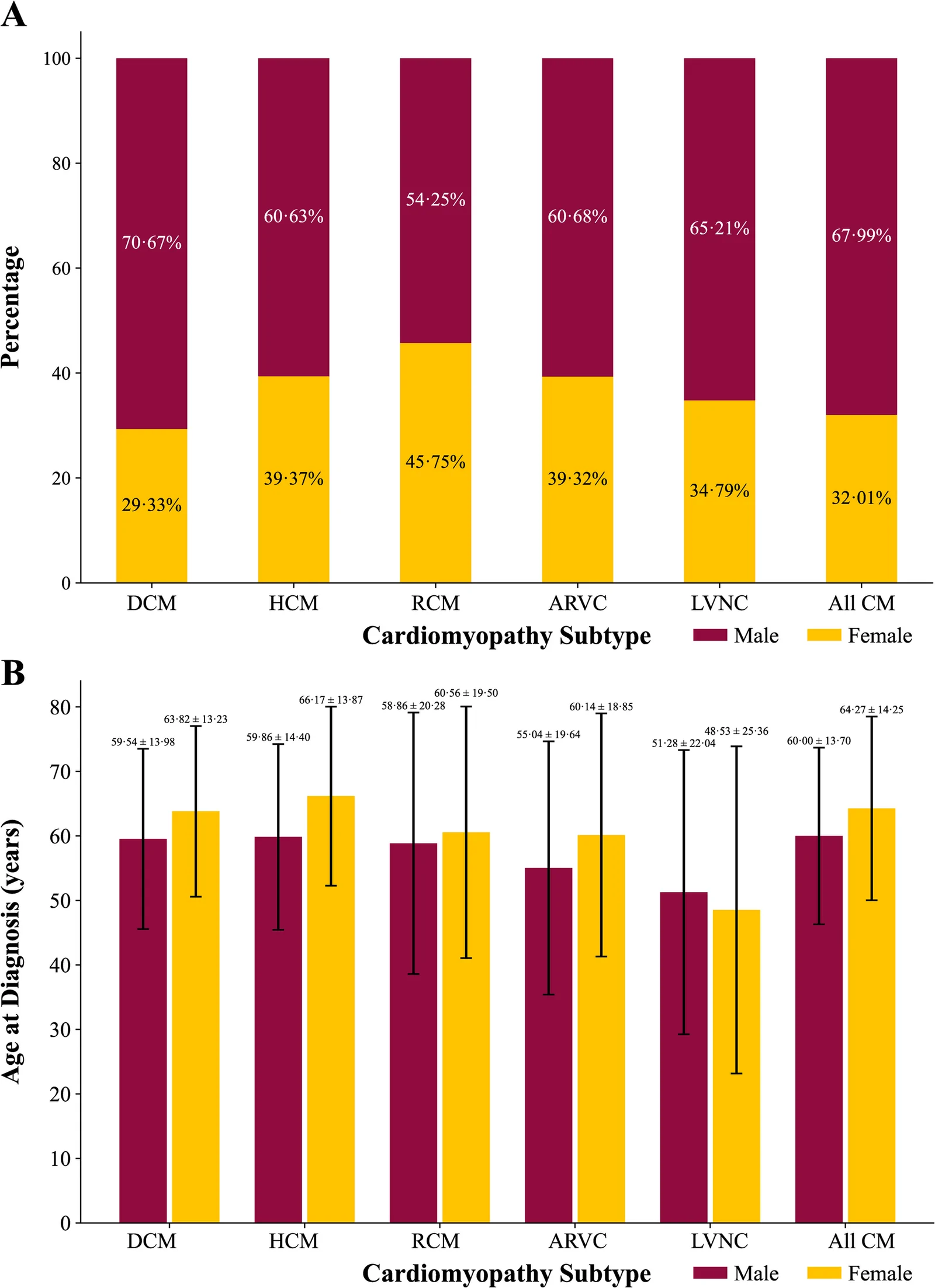
Demographic characteristics of patients with different types of cardiomyopathies. **A** Sex of patients with cardiomyopathies. Sex distribution is presented for each type of cardiomyopathy. **B** Age at diagnosis of patients with cardiomyopathies. Age at diagnosis is presented as mean ± standard deviation for each type of cardiomyopathy and sex. ARVC: arrhythmogenic right ventricular cardiomyopathy, CM: cardiomyopathies, DCM: dilated cardiomyopathy, HCM: hypertrophic cardiomyopathy, LVNC: left ventricular noncompaction, RCM: restrictive cardiomyopathy

There was a notable male predominance across all cardiomyopathy cases, with males constituting 67.99% of the patient population. This disparity was most pronounced among patients with DCM (70.67% male) and those with LVNC (65.21% male) and least pronounced among those with RCM (54.25% male; Fig. 2A).

The mean age at diagnosis for all cardiomyopathies combined was 60.77 ± 14.45 years. Men were diagnosed earlier (60.0 ± 13.7 years) than were women (64.3 ± 14.2 years). The youngest mean age at diagnosis was noted for LVNC (51.3 ± 22.0 years for men, 48.5 ± 25.4 years for women), whereas the oldest was observed for HCM (59.9 ± 14.4 years for men, 66.2 ± 13.9 years for women). Notably, women were consistently diagnosed at an older age than were men across all types of cardiomyopathies except LVNC (Fig. 2B).

### Hospitalization rates of patients with cardiomyopathies

From 2018 to 2022, the overall age- and sex-standardized hospitalization rate for cardiomyopathies in China increased from 151.55 to 206.23 per 1,000,000 population, with an AAPC of 7.68%. Regarding specific types, DCM consistently exhibited the highest hospitalization rate throughout the study period, followed by HCM, LVNC, RCM, and ARVC. Notably, ARVC and LVNC showed the most rapid increases, with AAPCs of 31.70% and 23.24%, respectively. The hospitalization rates for RCM remained low, with no significant change overall. However, there was a significant increasing trend in the hospitalization rate for RCM from 2018 to 2020, followed by an insignificant decrease from 2020 to 2022 (Table 1).

**Table 1:** Trends of hospitalization rates of patients with cardiomyopathies in China, 2018–2022

Diagnosis	Sex	Hospitalization rate					Trend		Trend – jointpoints	
		2018	2019	2020	2021	2022	AAPC	*p*	[Period 1] APC (95% CI) *p*	[Period 2] APC (95% CI) *p*
DCM	Male	157.90 (157.61–158.19)	182.81 (182.50–183.12)	177.58 (177.27–177.89)	201.12 (200.79–201.45)	201.42 (201.09–201.75)	6.00 (2.91–9.07)	< 0.0001		
	Female	70.97 (70.77–71.17)	81.28 (81.07–81.49)	77.91 (77.70–78.12)	85.67 (85.45–85.89)	84.61 (84.39–84.83)	4.12 (0.533–7.69)	0.02		
	Total	115.31 (115.13–115.49)	133.03 (132.84–133.22)	128.70 (128.51–128.89)	144.54 (144.34–144.74)	144.18 (143.98–144.38)	5.44 (2.24–8.62)	< 0.0001		
HCM	Male	38.47 (38.33–38.61)	50.14 (49.98–50.30)	49.37 (49.21–49.53)	61.57 (61.39–61.75)	64.31 (64.13–64.50)	13.12 (7.47–18.72)	< 0.0001		
	Female	27.27 (27.15–27.39)	34.92 (34.78–35.06)	33.78 (33.64–33.92)	40.89 (40.74–41.04)	42.72 (42.57–42.87)	11.13 (4.79–17.53)	< 0.0001		
	Total	32.97 (32.88–33.06)	42.69 (42.58–42.80)	41.76 (41.65–41.87)	51.46 (51.34–51.58)	53.73 (53.61–53.85)	12.34 (6.47–18.20)	< 0.0001		
RCM	Male	0.80 (0.78–0.82)	1.03 (1.01–1.05)	0.97 (0.95–0.99)	0.91 (0.89–0.93)	0.94 (0.92–0.96)	2.54 (-3.33–7.82)	0.43	[2018–2020] 16.70 (-0.94–38.02) *p* = 0.06	[2020–2022] -5.12 (-21.80–5.89) *p* = 0.18
	Female	0.75 (0.73–0.77)	0.81 (0.79–0.83)	0.83 (0.81–0.85)	0.85 (0.83–0.87)	0.82 (0.80–0.84)	2.08 (0.82–3.58)	< 0.0001	[2018–2020] 5.35 (3.57–11.13) *p* < 0.0001	[2020–2022] -3.15 (-7.17–0.59) *p* = 0.12
	Total	0.77 (0.76–0.78)	0.92 (0.90–0.94)	0.90 (0.88–0.92)	0.88 (0.86–0.90)	0.87 (0.85–0.89)	2.43 (-1.01–5.41)	0.19	[2018–2020] 13.35 (4.08–24.88) *p* = 0.01	[2020–2022] -3.61 (-14.59–0.93) *p* = 0.10
ARVC	Male	0.67 (0.65–0.69)	0.70 (0.68–0.72)	0.80 (0.78–0.82)	1.45 (1.42–1.48)	1.60 (1.57–1.63)	27.06 (12.50–44.60)	< 0.0001	[2018–2020] 5.60 (-18.55–58.72) *p* = 0.60	[2020–2022] 41.99 (-0.60–95.48) *p* = 0.06
	Female	0.40 (0.39–0.42)	0.37 (0.36–0.38)	0.57 (0.55–0.59)	1.05 (1.03–1.07)	1.15 (1.12–1.18)	37.10 (-2.58–92.43)	0.07		
	Total	0.53 (0.52–0.54)	0.54 (0.53–0.55)	0.69 (0.68–0.70)	1.25 (1.23–1.27)	1.38 (1.36–1.40)	31.70 (1.86–69.96)	0.03		
LVNC	Male	2.18 (2.15–2.21)	2.96 (2.92–3.00)	3.47 (3.43–3.51)	5.08 (5.03–5.13)	4.79 (4.74–4.84)	22.91 (15.70–31.25)	< 0.0001	[2018–2020] 32.78 (16.24–65.35) *p* < 0.0001	[2020–2022] 8.07 (-11.43–30.83) *p* = 0.32
	Female	1.25 (1.22–1.28)	1.62 (1.59–1.65)	1.93 (1.90–1.96)	2.82 (2.78–2.86)	2.65 (2.61–2.69)	22.84 (14.55–31.49)	< 0.0001		
	Total	1.72 (1.70–1.74)	2.31 (2.28–2.34)	2.71 (2.68–2.74)	3.97 (3.94–4.00)	3.73 (3.70–3.76)	23.24 (12.49–34.61)	< 0.0001		
All CM	Male	201.22 (200.89–201.55)	239.38 (239.02–239.74)	234.35 (234.00–234.70)	273.52 (273.14–273.90)	277.08 (276.70–277.46)	8.04 (4.37–11.72)	< 0.0001		
	Female	100.35 (100.11–100.59)	118.81 (118.55–119.07)	115.21 (114.96–115.46)	131.83 (131.56–132.10)	132.95 (132.68–133.22)	6.89 (3.02–10.71)	< 0.0001		
	Total	151.55 (151.35–151.75)	180.05 (179.83–180.27)	175.73 (175.51–175.95)	203.84 (203.60–204.08)	206.23 (205.99–206.47)	7.68 (4.18–11.22)	< 0.0001		

Hospitalization rate was measured per 1,000,000 people. AAPC is expressed as a percentage (%). APC is expressed as a percentage (%). Rates are expressed as patient-year incidence per 1,000,000 population (year-deduplicated patient counts as numerator). Trend segments were identified by the Joinpoint Regression Program using Bayesian Information Criterion model selection with a maximum of 1 joinpoint. Visual representations of these temporal trends are provided as line graphs in Figure S2

*AAPC* Average annual percentage change, *APC* Annual percentage change, *ARVC* Arrhythmogenic right ventricular cardiomyopathy, *CI* Confidence interval, *CM* Cardiomyopathy, *DCM* Dilated cardiomyopathy, *HCM* Hypertrophic cardiomyopathy, *LVNC* Left ventricular noncompaction, *RCM* Restrictive cardiomyopathy

Men consistently had higher hospitalization rates than women across all types of cardiomyopathies. In 2022, the overall hospitalization rate for men (277.08 per 1,000,000 population) was more than twofold that for women (132.95 per 1,000,000 population). This sex disparity was most pronounced in DCM, where the male rate (201.42 per 1,000,000 population) considerably exceeded the female rate (84.61 per 1,000,000 population). In contrast, RCM exhibited the most balanced sex distribution, with hospitalization rates of 0.94 and 0.82 per 1,000,000 population for men and women, respectively, in 2022 (Table 1).

**Fig. 3:**
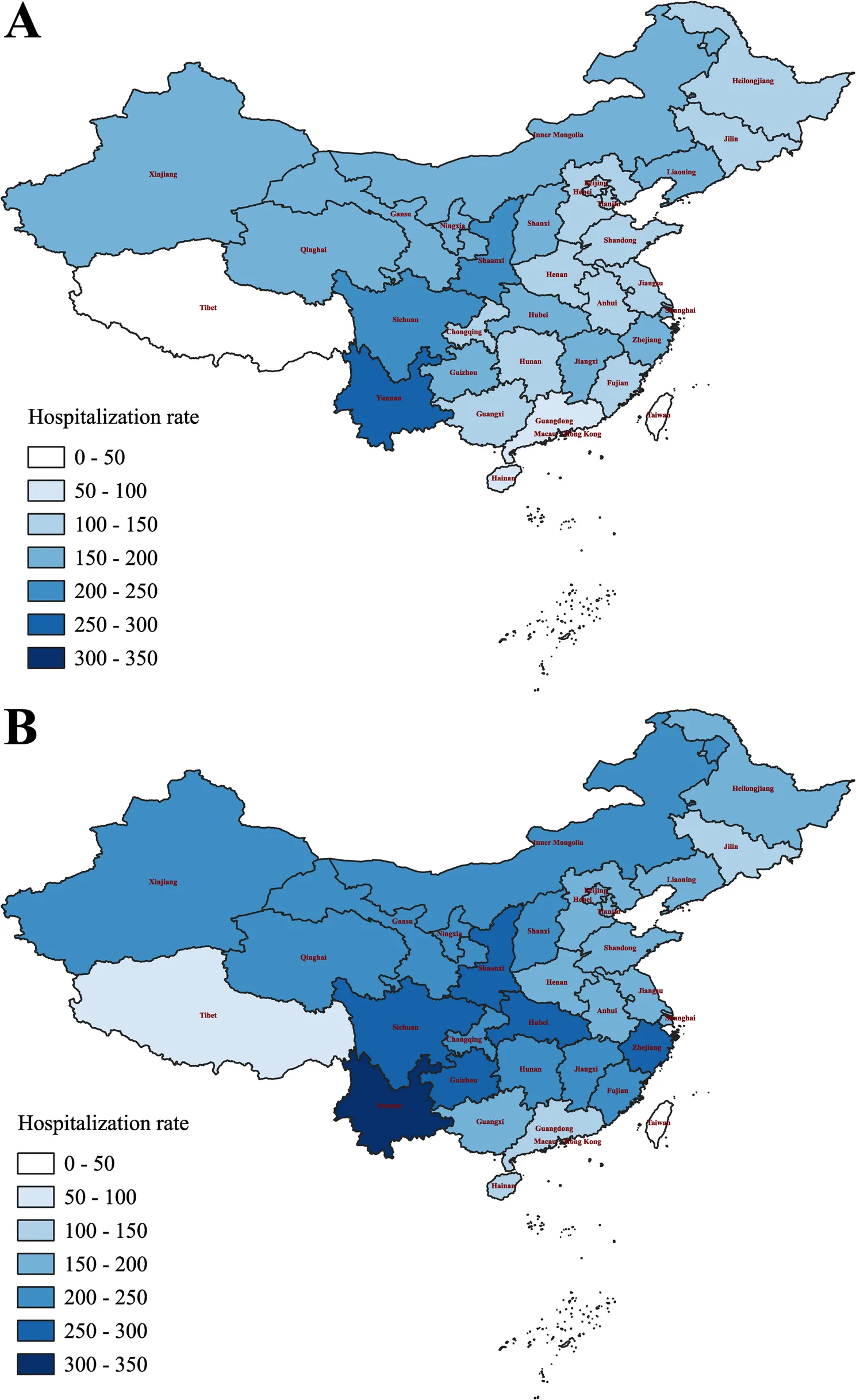
Regional variations and temporal changes in cardiomyopathy hospitalization rates in China, 2018–2022. Geographical distribution of cardiomyopathy hospitalization rates across China in (**A**) 2018 and (**B**) 2022. The color intensity represents the hospitalization rate per 1,000,000 population, with darker shades indicating higher rates

Regional variations in cardiomyopathy/hospitalization rates were evident across China (Fig. 3). Most regions experienced increases in hospitalization rates from 2018 to 2022, with Guizhou showing the most prominent increase (87.25%), followed by Hunan (79.69%), and Tibet (65.71%). Conversely, Jilin and Shanghai exhibited slight decreases of 1.51% and 4.43%, respectively. The detailed hospitalization rates for each region are provided in Table S2.

### Standardization to national hospital admissions

Cardiomyopathy hospitalizations were additionally standardized against national hospital admission counts (Fig. 1B). The standardized rate increased monotonically across the study period, from 158.83 to 215.39 per 100,000 national hospital admissions (cumulative + 35.6%). In 2020, when national hospital admissions decreased by 13.4% relative to 2019, the standardized cardiomyopathy proportion increased by 10.1% (from 182.49 to 200.96 per 100,000).

### Clinical characteristics of patients with cardiomyopathies

Among comorbidities, heart failure was the most prevalent (average 73.23% across all CM types), followed by arrhythmia (44.29%) and hypertension (39.95%). Heart failure was most prevalent in patients with DCM (average 83.71%) and least in patients with HCM (42.54%). Arrhythmias were most common in patients with ARVC (average 72.12%) and least in those with HCM (36.18%). Hypertension was the most prevalent in patients with HCM (average 58.39%) and least in patients with ARVC (26.14%). Stroke prevalence was highest in patients with HCM (average 15.24%) and lowest in those with LVNC (7.00%) (Table 2).

**Table 2:** Clinical characteristics of patients with cardiomyopathies

	CM	2018 (%)	2019 (%)	2020 (%)	2021 (%)	2022 (%)	Average (%)
Comorbidity							
Heart failure	DCM	77.58	80.71	83.79	86.71	88.31	83.71
	HCM	41.38	42.01	43.12	42.96	42.83	42.54
	RCM	70.72	76.83	74.96	80.42	80.28	76.77
	ARVC	48.04	56.58	56.98	51.69	53.68	53.29
	LVNC	69.01	71.10	71.25	63.54	71.09	68.80
	All CM	69.46	71.28	73.72	74.85	75.68	73.23
Arrhythmia	DCM	43.20	44.85	46.91	48.76	49.46	46.81
	HCM	33.97	34.72	36.47	36.85	37.84	36.18
	RCM	56.27	59.61	57.28	61.08	59.98	58.90
	ARVC	64.79	81.32	82.24	71.07	67.30	72.12
	LVNC	40.81	41.88	42.73	40.49	44.44	42.19
	All CM	41.32	42.58	44.53	45.69	46.39	44.29
Hypertension	DCM	31.43	33.20	34.69	35.63	35.94	34.30
	HCM	55.38	57.03	58.44	59.70	60.03	58.39
	RCM	24.06	27.49	28.01	28.89	30.93	27.96
	ARVC	16.23	21.71	24.02	28.59	30.59	26.14
	LVNC	24.78	29.98	29.3	30.89	28.49	29.10
	All CM	36.45	38.71	40.13	41.47	41.94	39.95
Stroke	DCM	6.81	7.24	7.79	7.96	8.20	7.64
	HCM	14.04	14.90	15.63	15.66	15.98	15.24
	RCM	8.14	9.42	8.84	8.67	10.55	9.33
	ARVC	6.41	3.55	5.65	11.02	13.32	7.99
	LVNC	5.75	6.97	6.33	8.03	7.81	7.00
	All CM	8.36	9.04	9.62	9.91	10.24	9.43
Diabetes	DCM	14.01	15.06	16.03	16.99	17.71	16.06
	HCM	18.32	19.51	20.45	21.68	18.32	19.72
	RCM	11.71	12.59	14.63	14.37	15.75	13.85
	ARVC	8.12	8.68	11.50	9.60	10.85	9.99
	LVNC	11.26	12.49	12.12	13.81	13.05	12.78
	All CM	14.76	15.75	16.75	17.71	18.55	16.84
CKD	DCM	4.21	4.60	5.44	6.04	6.95	5.52
	HCM	3.70	3.78	4.46	4.96	5.46	4.57
	RCM	5.95	6.64	7.98	7.78	8.69	7.44
	ARVC	1.70	3.55	3.70	2.99	3.08	3.04
	LVNC	3.90	4.48	5.29	5.25	5.79	5.11
	All CM	4.12	4.44	5.26	5.83	6.61	5.34
Liver disease	DCM	2.62	3.00	4.69	6.05	7.51	4.90
	HCM	1.01	1.28	1.94	2.57	3.10	2.10
	RCM	3.75	3.63	4.93	6.42	6.98	5.16
	ARVC	2.75	2.37	4.11	4.41	4.37	3.90
	LVNC	2.30	1.96	3.42	4.16	6.03	3.93
	All CM	2.27	2.58	4.02	5.14	6.30	4.21
Procedure							
Pacemaker	DCM	2.87	2.88	2.89	2.81	2.86	2.86
	HCM	2.95	2.88	3.04	2.88	2.97	2.94
	RCM	5.12	5.33	6.1	4.57	4.63	5.16
	ARVC	5.89	8.16	8.52	6.27	6.48	6.87
	LVNC	2.47	2.45	2.32	2.11	2.72	2.40
	All CM	2.9	2.91	2.96	2.86	2.92	2.91
ICD	DCM	0.64	0.74	0.86	1.1	1.27	0.94
	HCM	0.37	0.37	0.4	0.41	0.48	0.41
	RCM	0.18	0.23	0.47	0.32	0.32	0.31
	ARVC	4.32	6.18	7.7	5.03	5.71	5.71
	LVNC	0.7	1.14	1.07	0.89	1.17	1.01
	All CM	0.59	0.68	0.78	0.94	1.09	0.83

Percentages (%) represent the proportion of patients with each comorbidity or procedure among all patients hospitalized for a specific type of cardiomyopathy in a given year

*ARVC* Arrhythmogenic right ventricular cardiomyopathy, *CKD* Chronic kidney disease, *CM* Cardiomyopathy, *DCM* Dilated cardiomyopathy, *HCM* Hypertrophic cardiomyopathy, *ICD* Implantable cardioverter-defibrillator, *LVNC* Left ventricular noncompaction, *RCM* Restrictive cardiomyopathy

The prevalence of non-cardiovascular comorbidities, such as diabetes, chronic kidney disease (CKD), and liver disease, was relatively low overall (averaging 16.84%, 5.34%, and 4.21% across all CM types, respectively). Patients with DCM generally exhibited the highest average prevalence of these conditions, while patients with ARVC had the lowest (Table 2).

Pacemaker implantation was most common in patients with ARVC (average 6.87%) and least in those with LVNC (2.40%). Implantable cardioverter-defibrillator (ICD) use was highest in patients with ARVC (average 5.71%) and lowest in patients with RCM (0.31%) (Table 2).

### Inpatient outcomes and resource utilization of patients with cardiomyopathies

The percentage of patients admitted to the intensive care unit increased significantly from 2.49% in 2018 to 6.97% in 2022 (AAPC = 33.94%, *p* = 0.01). Among specific cardiomyopathy types, ARVC exhibited the highest increase, increasing from 2.36% to 9.20% (AAPC = 41.13%, *p* = 0.04), notably between 2018 and 2020 (APC = 91.96%, *p* < 0.0001). DCM also showed a significant increase in intensive care unit admissions from 2.46% to 6.72% (AAPC = 31.36%, *p* < 0.0001; Table 3).

**Table 3:** Trends of inpatient outcomes and resource utilization of patients with cardiomyopathies, 2018–2022

CM		2018	2019	2020	2021	2022	AAPC	*p*	[Period 1] APC (95% CI) *p*	[Period 2] APC (95% CI) *p*
DCM	Admission to ICU (%)	2.46	2.8	5.35	6.2	6.72	31.36 (13.06–53.57)	< 0.0001	[2018–2020] 47.45 (5.09–147.26) *p* = 0.02	[2020–2022] 8.35 (-32.62–64.42) *p* = 0.52
	In-hospital death (%)	0.87	0.93	0.91	0.81	0.90	-0.70 (-4.47–3.13)	0.72		
	Length (days)	8.00 (6.00, 11.00)	8.00 (6.00, 11.00)	8.00 (6.00, 11.00)	8.00 (6.00, 10.00)	7.00 (5.00, 10.00)	-2.91 (-5.26 to -0.18)	0.03	[2018–2020] 1.27 (-2.96–11.99) *p* = 0.34	[2020–2022] -9.50 (-16.57 to -2.63) *p* = 0.01
	Costs (CNY)	7,061.20 (4,568.51, 11,177.00)	7,408.06 (4,777.27, 11,749.51)	7,443.34 (4,831.72, 11,743.82)	7,800.87 (5,045.76, 12,187.34)	7,649.41 (4,965.44, 11,944.79)	2.14 (-0.63–4.94)	0.14		
HCM	Admission to ICU (%)	2.52	2.51	5.86	7.11	7.23	37.01 (-2.23–91.49)	0.08		
	In-hospital death (%)	0.74	0.69	0.76	0.72	0.78	1.49 (-1.65–4.61)	0.40		
	Length (days)	8.00 (5.00, 11.00)	7.00 (5.00, 11.00)	7.00 (5.00, 11.00)	7.00 (5.00, 10.00)	7.00 (5.00, 10.00)	-2.91 (-4.62 to -0.81)	< 0.0001	[2018–2020] -9.50 (-13.61 to -3.32) *p* < 0.0001	[2020–2022] 1.27 (-2.23–7.46) *p* = 0.34
	Costs (CNY)	8,412.53 (5,268.68, 13,968.56)	8,606.59 (5,357.81, 14,734.51)	8,736.32 (5,401.02, 15,503.38)	9,072.83 (5,667.34, 16,185.55)	8,768.76 (5,558.10, 15,603.10)	1.27 (0.45–2.19)	< 0.0001	[2018–2020] 2.76 (1.22–5.77) *p* = 0.004	[2020–2022] -1.17 (-3.67–1.50) *p* = 0.52
RCM	Admission to ICU (%)	3.39	2.93	7.12	8.43	7.87	31.54 (-2.77–76.41)	0.08		
	In-hospital death (%)	2.56	1.62	2.19	2.33	2.52	2.42 (-9.22–17.09)	0.60	[2018–2020] -18.84 (-40.09–21.49) *p* = 0.31	[2020–2022] 17.76 (-15.22–65.62) *p* = 0.21
	Length (days)	9.00 (6.00, 14.00)	9.00 (6.00, 14.00)	10.00 (6.00, 14.75)	9.00 (6.00, 13.00)	8.00 (6.00, 12.00)	-2.33 (-9.92–5.90)	0.42	[2018–2020] 5.69 (2.27–14.33) *p* < 0.0001	[2020–2022] -9.73 (-16.65 to -6.70) *p* < 0.0001
	Costs (CNY)	9,879.73 (5,839.8, 18,626.34)	10,687.18 (6,165.58, 19,207.12)	10,504.32 (5,849.12, 20,796.93)	9,595.14 (5,748.64, 17,528.6)	9,500.19 (5,717.22, 17,149.81)	-1.56 (-4.55–1.05)	0.20	[2018–2020] 5.75 (-2.77–15.05) *p* = 0.18	[2020–2022] -5.70 (-15.20 to -0.66) *p* = 0.03
ARVC	Admission to ICU (%)	2.36	4.47	8.83	8.59	9.20	41.13 (3.10–90.46)	0.04	[2018–2020] 91.96 (83.67–100.76) *p* < 0.0001	[2020–2022] 2.30 (-2.29–6.84) *p* = 0.32
	In-hospital death (%)	0.65	1.05	0.62	0.85	1.29	12.29 (-24.43–65.20)	0.46		
	Length (days)	7.00 (5.00, 11.00)	7.50 (5.00, 11.00)	7.00 (5.00, 11.00)	7.00 (5.00, 10.00)	7.00 (5.00, 10.00)	-0.69 (-2.59–1.21)	0.45		
	Costs (CNY)	7,992.55 (5,029.32, 15,681.43)	9,565.88 (5,342.18, 29,029.54)	9,355.43 (5,547.74, 29,651.08)	7,839.70 (4,942.71, 16,242.81)	7,421.88 (4,668.33, 15,214.30)	-2.75 (-8.30–2.03)	0.21	[2018–2020] 15.26 (-0.24–35.00) *p* = 0.06	[2020–2022] -12.18 (-28.73 to -5.51) *p* = 0.004
LVNC	Admission to ICU (%)	3.25	3.22	8.81	8.89	9.18	36.24 (-2.78–87.91)	0.08		
	In-hospital death (%)	0.82	0.71	0.73	0.75	0.68	-3.15 (-11.87–6.21)	0.39		
	Length (days)	8.00 (6.00, 12.00)	8.00 (6.00, 12.00)	8.00 (5.00, 11.00)	7.00 (5.00, 10.00)	7.00 (5.00, 10.00)	-3.93 (-8.23–0.37)	0.07		
	Costs (CNY)	9,098.19 (5,623.21, 16,355.29)	9,273.87 (5,685.37, 16,158.95)	9,489.83 (5,791.76, 16,870.81)	10,620.34 (6,384.36, 17,678.07)	9,553.95 (6,014.84, 16,972.72)	2.36 (-0.75–5.54)	0.14		
All CM	Admission to ICU (%)	2.49	2.75	5.57	6.52	6.97	33.94 (5.35–68.84)	0.01		
	In-hospital death (%)	0.85	0.88	0.89	0.80	0.89	-0.03 (-3.45–3.41)	0.96		
	Length (days)	8.00 (6.00, 11.00)	8.00 (6.00, 11.00)	8.00 (6.00, 11.00)	8.00 (5.00, 10.00)	7.00 (5.00, 10.00)	-2.91 (-5.26–0.18)	0.03	[2018–2020] 1.27 (-2.96–11.99) *p* = 0.37	[2020–2022] -9.50 (-16.57 to -2.63) *p* = 0.01
	Costs (CNY)	7,362.55 (4,709.18, 11,816.06)	7,711.43 (4,913.29, 12,457.48)	7,782.54 (4,968.85, 12,589.46)	8,174.94 (5,217.42, 13,154.71)	7,985.18 (5,128.52, 12,877.95)	2.13 (0.15–4.27)	0.03	[2018–2020] 3.61 (-0.72–11.02) *p* = 0.11	[2020–2022] -0.28 (-6.40–5.20) *p* = 0.86

AAPC was expressed as a percentage (%). APC is expressed as a percentage (%). All metrics are computed under the pre-specified patient-year analytic framework (one record per patient per calendar year, retaining the first admission of the year). Trend segments were identified by the Joinpoint Regression Program using Bayesian Information Criterion model selection with a maximum of 1 joinpoint. Visual representations of these temporal trends are provided as line graphs in Figure S3

*AAPC* Average annual percentage change, *APC* Average annual percent change, *ARVC* Arrhythmogenic right ventricular cardiomyopathy, *CM* Cardiomyopathy, *CNY* Chinese Yuan, *DCM* Dilated cardiomyopathy, *HCM* Hypertrophic cardiomyopathy, *ICU* Intensive care unit, *LVNC* Left ventricular noncompaction, *RCM* Restrictive cardiomyopathy

Despite increased intensive care unit admissions, in-hospital mortality rates remained stable, at 0.85% in 2018 and at 0.89% in 2022 (AAPC = − 0.03%, *p* = 0.96). Notably, patients with RCM had the highest mortality rates, averaging around 2.5% throughout the study period (Table 3).

The median length of hospital stay decreased slightly, from 8 days (IQR: 6–11 days) in 2018 to 7 days (IQR: 5–10 days) in 2022 (AAPC = − 2.91%, *p* = 0.03). This reduction was primarily observed among patients with HCM and DCM (Table 3).

The median hospitalization cost increased slightly, from 7,362.55 Chinese Yuan (CNY; IQR: 4,709.18–11,816.06 CNY) in 2018 to 7,985.18 CNY (IQR: 5,128.52–12,877.95 CNY) in 2022 (AAPC = 2.13%, *p* = 0.03). Among cardiomyopathy types, only HCM showed a significant cost increase (AAPC = 1.27%, *p* < 0.0001; Table 3).

## Discussion

This comprehensive, nationwide analysis of hospitalized patients with cardiomyopathies in China from 2018 to 2022, utilizing the HQMS database, reveals significant insights into the evolving epidemiological landscape and clinical management of these disorders. We observed an increasing trend in overall hospitalization rates for cardiomyopathies. This period notably followed the publication of the First China Rare Disease List in 2018, which included DCM, HCM, RCM, ARVC, and LVNC. While this policy initiative and subsequent efforts aimed to enhance recognition, diagnosis, and management, our study provides a broad assessment of the real-world hospitalization patterns and patient characteristics on a national scale.

The rising hospitalization rates are a positive indicator, likely reflecting advancements in diagnostic capabilities and clinical awareness rather than solely an increase in disease incidence. This trend, particularly after the 2018 Rare Disease List inclusion, suggests national policy initiatives have enhanced disease recognition and access to specialized care. This is supported by the fact that China’s hospitalization rates remain significantly lower than those in developed countries, indicating a substantial gap in recognition and management. For instance, in 2022, China’s rates per million were 144.18 for DCM and 53.73 for HCM, compared to a French study in 2015 reporting 472.8 and 101.7, respectively [9]. Similarly, a 2018 UK study found rates of 430 for DCM and 350 for HCM [10]. While most tertiary hospitals in China have basic diagnostic capabilities, a nationwide survey reported suboptimal availability of specialized tools like CMR and EMB [11]. These disparities underscore the urgent need to improve disease awareness, diagnostic accuracy, and access to care. Although China has made significant efforts through clinical networks, multidisciplinary care, and standardized guidelines since 2018 [6], our findings suggest these initiatives have yet to achieve full nationwide implementation.

An important methodological consideration is whether the observed temporal trend was confounded by pandemic-era changes in overall hospital utilization. Standardization against national hospital admission counts speaks to this concern: the proportion of cardiomyopathy hospitalizations among all national hospital admissions increased every year from 2018 to 2022, including a 10.1% rise during the peak pandemic year of 2020 when total national admissions contracted by 13.4%. The apparent attenuation of absolute counts in 2020 thus appears to reflect generalized healthcare access reduction rather than a slowdown in cardiomyopathy hospitalization. The clinical plausibility of this counter-cyclical pattern lies in the nature of cardiomyopathy inpatient care, which typically reflects initial diagnostic work-up, episodes of clinical decompensation, or procedural interventions such as cardiac device implantation — categories that are inherently less deferrable than the elective and lower-acuity admissions that constituted the majority of pandemic-era workload reduction. This pattern is consistent with the interpretation that the observed increase reflects genuine improvement in disease recognition and specialized care pathways rather than secular growth in overall hospital utilization.

Our study highlights the importance of focusing on the distribution of cardiomyopathy types. DCM consistently had the highest hospital-based prevalence, followed by HCM. Despite population studies suggesting a higher incidence of HCM than DCM, the proportion of HCM patients requiring hospitalization was significantly lower, aligning with previous research [11]. The higher hospitalization rate for DCM is attributable to more frequent and severe cardiovascular complications, such as heart failure and arrhythmias, often requiring invasive interventions [12]. These findings underscore the need for targeted interventions in DCM, including early diagnosis and prompt referral for advanced therapies.

Less common cardiomyopathies like ARVC and LVNC showed higher hospitalization growth rates than DCM and HCM, a trend that mirrors findings from UK and French studies [9, 10]. This suggests that growing awareness contributes to this growth beyond incidence rates.

The trajectory of RCM hospitalization activity provides a clinically informative example of how disease-modifying therapy availability can drive diagnostic awareness for rare cardiomyopathy phenotypes. The most pronounced single-year change in annual RCM hospitalizations occurred between 2018 and 2019 (+ 18.5%), coinciding with the US Food and Drug Administration’s May 2019 approval of tafamidis — the first disease-modifying therapy for transthyretin amyloid cardiomyopathy, a major substrate of the RCM phenotype — which catalyzed global expansion of cardiac amyloidosis diagnostic awareness and bone-scintigraphy uptake. Annual RCM counts subsequently plateaued slightly below the 2019 peak during 2020–2022, likely influenced by COVID-19-era disruptions and by the comparatively late National Medical Products Administration (NMPA) approval of tafamidis in China (December 2021).

Analysis of regional variations revealed heterogeneous distributions, with most regions experiencing increasing trends but with considerable variability in magnitude. The disproportionately large percentage changes observed in Guizhou (+ 87.25%) and Tibet (+ 65.71%) should be interpreted with caution: these provinces have smaller absolute baseline cohorts, so modest absolute increments translate into amplified relative changes. These percentage gains also likely reflect expanding rare-disease diagnostic capacity and improving healthcare access in historically underserved western and inland regions following the 2018 inclusion of primary cardiomyopathies in the National Rare Disease List. Beyond the small-baseline effect, three categories of drivers likely contribute to the broader pattern of regional variation. First, healthcare-system supply-side factors — including the density of tertiary cardiovascular centers, availability of cardiac imaging (cardiac magnetic resonance and bone scintigraphy for amyloidosis), and the geographic distribution of rare-disease genetic counseling and testing services — remain concentrated in eastern coastal provinces and major metropolitan areas, contributing to higher diagnostic ascertainment in those regions. Second, demographic and population-structure factors, including regional differences in population aging, internal migration patterns, and the geographic distribution of high-prevalence comorbidities (hypertension and diabetes prevalence vary substantially across provinces), shape both the underlying disease burden and the case-presentation profile. Third, potential measurement bias may contribute to the observed disparities: regional variation in HQMS data-completeness, in adherence to NCV 2.0 coding standards, and in the institutional case-mix served by secondary-level hospitals could differentially affect ascertainment across provinces, particularly in regions where tertiary care is geographically concentrated and complex cases are referred across provincial boundaries. From a policy perspective, these findings underscore the need for region-specific strategies in China’s rare-disease healthcare-system development. Provinces with rising absolute cohorts despite small populations (such as Guizhou and Tibet) may benefit most from sustained investment in regional rare-disease diagnostic infrastructure, telemedicine linkage to national referral centers, and standardized coding training for inpatient discharge departments. Provinces with persistently low hospitalization rates may warrant targeted gap analyses to distinguish genuine low disease prevalence from under-ascertainment. This regional differentiation aligns with the broader objectives of China’s National Rare Disease Comprehensive Service System established following the 2018 First Rare Disease List.

The sex distribution showed a notable male predominance across all types, consistent with previous findings for DCM, HCM, and RCM [10]. The reasons for male predominance in HCM are unclear, with potential factors including differences in health-seeking behavior, hormonal influences, and environmental factors [13–17].

Men were diagnosed at a younger age than women, except for LVNC. While previous research on HCM consistently shows women are diagnosed older [13–18], limited data exist for other types. Our findings highlight the need for further research on the social and biological mechanisms underlying these sex differences in cardiomyopathies.

The clinical characteristics revealed complex management challenges. The high prevalence of cardiovascular comorbidities underscores the need for comprehensive management. Notably, frequent heart failure in DCM patients emphasizes the importance of early intervention. The highest stroke rates in HCM patients and high arrhythmia rates in ARVC patients are consistent with clinical practice and necessitate targeted interventions. The prevalence of hypertension exceeding 50% in the HCM group highlights the need to differentiate it from hypertension-induced cardiac hypertrophy [19]. The presence of other comorbidities like diabetes, CKD, and liver disease can also significantly impact outcomes.

Utilization of invasive cardiac electronic devices varied among subtypes. While pacemaker implantation rates remained relatively stable, the use of ICDs increased significantly. The rising uptake of ICDs, particularly in high-risk populations like patients with ARVC, highlights progress in risk stratification and adherence to guideline-recommended prevention strategies.

A key finding reflecting improved healthcare quality is that in-hospital mortality rates remained stable, which is particularly noteworthy given the significant increase in intensive care unit (ICU) admissions. This suggests hospitals are managing a more severely ill patient population. The combination of stable mortality amidst rising patient acuity strongly indicates a substantial improvement in the quality of acute care and the effectiveness of management strategies for critical complications.

Furthermore, improvements in healthcare efficiency are evident. The slight decrease in the median length of hospital stay, particularly for DCM and HCM, points towards more streamlined care protocols. Considering the national inflation rate (average 2.07% from 2018 to 2022), the minimal increase in hospitalization costs (AAPC = 2.13%) suggests these gains in management efficiency were achieved without a substantial rise in the financial burden, demonstrating enhanced resource utilization. Collectively, these metrics—stable mortality for more severe cases, reduced hospital stays, and controlled costs—paint a clear picture of an evolving and improving standard of care for cardiomyopathy patients in China.

### Limitations

Our study has several limitations. First, because the data do not cover primary healthcare facilities, our findings may have limited generalizability. This limitation is likely mitigated, however, as patients with cardiomyopathies are predominantly managed in higher-level hospitals under China’s hierarchical medical system. Second, the 5-year study period may limit the identification of long-term trends, though it was specifically chosen to assess the impact of the 2018 First China Rare Disease List. Future research with longer follow-up is needed to validate these trends. Third, the potential for misclassification and coding errors exists, although review processes were implemented to minimize such issues. Fourth, the pre-specified patient-year analytic framework retained each patient’s first admission per calendar year as the analytic unit. Consequently, estimates of length of stay, hospitalization cost, and in-hospital mortality reflect the index annual admission rather than cumulative annual experience, and may not fully represent the resource burden or outcomes for patients with subsequent within-year readmissions. The relative comparisons between subtypes, regions, and time periods that constitute the principal claims of this study are not materially affected, as the same patient-year definition is applied uniformly across all comparison groups. Fifth, cases were identified by cardiomyopathy codes appearing in either the principal or any secondary diagnostic position. While this approach was methodologically necessary to capture admissions driven by acute cardiac complications, it introduces the theoretical risk of capturing hospitalizations where cardiomyopathy is an incidental comorbidity, potentially influencing resource-utilization metrics. This risk is partially addressed by the cohort’s clinical profile — a high concurrent prevalence of heart failure (73.23%), arrhythmias (44.29%), and stroke (9.43%) — and by the contextual finding that 82.3% of these hospitalizations were managed within cardiac or other relevant medical departments (Supplementary Table S3). Sixth, data on advanced cardiovascular interventions — heart transplantation, ventricular assist device implantation, and extracorporeal membrane oxygenation — are beyond the scope of this diagnosis-focused epidemiological analysis. Future studies leveraging the China Heart Transplant Registry, the China Mechanical Circulatory Support Registry, and the National Center for Cardiovascular Diseases could address this dimension of cardiomyopathy resource utilization.

## Conclusions

Our findings provide valuable epidemiological and clinical insights into cardiomyopathies in China. The increased hospitalization rates following their inclusion in the First China Rare Disease List likely reflect improved awareness, diagnosis, and access to care. Nevertheless, lower hospitalization rates compared to developed countries, alongside persistent regional disparities, suggest ongoing challenges in early detection and equitable access to advanced therapies. Addressing these imbalances and insufficiencies requires expanding healthcare infrastructure, investing in specialized care and workforce training, and conducting public health campaigns. Furthermore, nationwide integration of genetic testing and multidisciplinary care models is needed to enhance diagnostic accuracy, complemented by sex- and age-specific strategies to address factors influencing disease progression. The experiences from China may offer valuable lessons for other developing countries managing complex cardiovascular conditions.

## Supplementary Information

Supplementary Material 1.

## Data Availability

The data that support the findings of this study are not publicly available. The data were obtained from China’s Hospital Quality Monitoring System (HQMS) under strict national regulations for health information security. Access to the dataset is restricted and requires a formal application and approval process. Qualified investigators with legitimate research proposals may contact the corresponding authors to initiate an inquiry for data access, which is subject to approval from the relevant national health authorities and ethics committees.

## Abbreviations

AAPC: Average annual percentage change
APC: Annual percentage change
ARVC: Arrhythmogenic right ventricular cardiomyopathy
CI: Confidence interval
CKD: Chronic kidney disease
CM: Cardiomyopathy
CMR: Cardiac magnetic resonance
CNY: Chinese Yuan
DCM: Dilated cardiomyopathy
EMB: Endomyocardial biopsy
HCM: Hypertrophic cardiomyopathy
HQMS: Hospital Quality Monitoring System
ICD: Implantable cardioverter-defibrillator
ICU: Intensive care unit
IQR: Interquartile range
LVNC: Left ventricular noncompaction
NCV 2.0: National Clinical Version 2.0
NMPA: National Medical Products Administration
RCM: Restrictive cardiomyopathy
STROBE: Strengthening the Reporting of Observational Studies in Epidemiology
