# Supplementary material for: Epidemiological and clinical characteristics of hospitalized patients with cardiomyopathies in China: a nationwide retrospective analysis
Source: BMC Public Health. 2026 Jul 10;26:2766. doi: 10.1186/s12889-026-28501-y (PMC13637130; doi:10.1186/s12889-026-28501-y)
Supplement: Supplementary file 1 [file 12889_2026_28501_MOESM1_ESM.docx]

**Supplemental Materials**

**Table S1**. National Clinical Version 2.0 codes for cardiomyopathies.

| **Disease** | **NCV 2.0 code** | **Code heading** |
| --- | --- | --- |
| DCM | I42.0 | Dilated cardiomyopathy |
| HCM | I42.1 | Obstructive hypertrophic cardiomyopathy |
|  | I42.2 | Other hypertrophic cardiomyopathy |
| RCM | I42.500x001 | Restrictive cardiomyopathy |
| ARVC | I42.800x002 | Arrhythmogenic right ventricular cardiomyopathy |
| LVNC | Q24.800x006 | Congenital myocardial noncompaction |
|  | Q24.806 | Ventricular myocardial noncompaction |
| Idiopathic cardiomyopathy | I42.900x003 | Idiopathic cardiomyopathy |

ARVC: arrhythmogenic right ventricular cardiomyopathy, DCM: dilated cardiomyopathy, HCM: hypertrophic cardiomyopathy, LVNC: left ventricular noncompaction, NCV: National Clinical Version, RCM: restrictive cardiomyopathy. The unspecified-cardiomyopathy code I42.900x003 is independent of the five phenotypic subtype codes; cases identified by I42.900x003 contribute only to the All-CM cohort count and were not analyzed within any subtype-specific sub-cohort.

**Table S2**. Regional cardiomyopathy hospitalization rates in China.

| **Region** | **Hospitalization rate (2018)** | **Hospitalization rate (2022)** |
| --- | --- | --- |
| Beijing | 135.52 | 165.39 |
| Tianjin | 121.74 | 162.20 |
| Hebei | 141.20 | 173.21 |
| Shanxi | 189.20 | 203.89 |
| Inner Mongolia | 197.60 | 213.69 |
| Liaoning | 155.95 | 165.81 |
| Jilin | 129.64 | 127.69 |
| Heilongjiang | 130.33 | 190.02 |
| Shanghai | 169.64 | 162.12 |
| Jiangsu | 136.63 | 181.25 |
| Zhejiang | 192.08 | 286.40 |
| Anhui | 118.85 | 154.11 |
| Fujian | 125.64 | 203.61 |
| Jiangxi | 158.18 | 209.50 |
| Shandong | 128.39 | 172.01 |
| Henan | 117.50 | 170.64 |
| Hubei | 177.52 | 277.34 |
| Hunan | 138.85 | 249.50 |
| Guangdong | 94.92 | 136.92 |
| Guangxi | 128.59 | 190.40 |
| Hainan | 86.50 | 124.59 |
| Chongqing | 146.63 | 221.50 |
| Sichuan | 203.27 | 288.75 |
| Guizhou | 150.87 | 282.51 |
| Yunnan | 264.29 | 325.55 |
| Tibet | 47.97 | 79.49 |
| Shaanxi | 209.47 | 283.49 |
| Gansu | 161.71 | 229.82 |
| Qinghai | 158.68 | 212.19 |
| Ningxia | 159.66 | 207.28 |
| Xinjiang | 167.30 | 217.78 |
| Taiwan | NA | NA |
| Hong Kong | NA | NA |
| Macau | NA | NA |

NA: Not available.

Hospitalization rates are expressed per 1,000,000 population.

**Table S3**. **Distribution of cardiomyopathy hospitalizations by discharge department and subtype in China.**

| Discharge department | All CM | DCM | HCM | RCM | ARVC | LVNC |
| --- | --- | --- | --- | --- | --- | --- |
| Departments clinically appropriate for managing acute cardiomyopathy complications | | | | | | |
| Cardiology | 706,188 (55.13) | 526,919 (56.82) | 154,692 (49.82) | 3,360 (55.42) | 3,871 (62.50) | 12,279 (60.46) |
| General internal medicine | 177,915 (13.89) | 143,806 (15.51) | 31,231 (10.06) | 571 (9.42) | 642 (10.36) | 1,132 (5.57) |
| Intensive care unit | 17,038 (1.33) | 12,498 (1.35) | 3,768 (1.21) | 105 (1.73) | 91 (1.47) | 337 (1.66) |
| Geriatrics | 16,780 (1.31) | 11,143 (1.20) | 4,952 (1.59) | 72 (1.19) | 57 (0.92) | 395 (1.94) |
| General practice | 18,601 (1.45) | 13,513 (1.46) | 4,658 (1.50) | 75 (1.24) | 60 (0.97) | 191 (0.94) |
| Subtotal | 936,522 (73.11) | 707,879 (76.33) | 199,301 (64.18) | 4,183 (68.99) | 4,721 (76.22) | 14,334 (70.58) |
| Cardiovascular-related departments managing cross-organ syndromes | | | | | | |
| Pulmonary medicine | 55,704 (4.35) | 43,533 (4.69) | 10,647 (3.43) | 190 (3.13) | 602 (9.72) | 505 (2.49) |
| Nephrology | 20,024 (1.56) | 13,896 (1.50) | 5,036 (1.62) | 112 (1.85) | 45 (0.73) | 280 (1.38) |
| Neurology | 42,608 (3.33) | 21,646 (2.33) | 20,177 (6.50) | 173 (2.85) | 83 (1.34) | 358 (1.76) |
| Subtotal | 118,336 (9.24) | 79,075 (8.53) | 35,860 (11.55) | 475 (7.83) | 730 (11.79) | 1,143 (5.63) |
| Cardiovascular relevant | 1,054,858 (82.34) | 786,954 (84.86) | 235,161 (75.73) | 4,658 (76.83) | 5,451 (88.00) | 15,477 (76.21) |
| Other departments | | | | | | |
| Gastroenterology | 23,020 (1.80) | 14,666 (1.58) | 7,918 (2.55) | 94 (1.55) | 82 (1.32) | 193 (0.95) |
| Other clinical departments | 203,146 (15.86) | 125,724 (13.56) | 67,452 (21.72) | 1,311 (21.62) | 661 (10.67) | 4,639 (22.84) |
| Subtotal | 226,166 (17.66) | 140,390 (15.14) | 75,370 (24.27) | 1,405 (23.17) | 743 (12.00) | 4,832 (23.79) |
| Total | 1,281,024 (100.0) | 927,344 (100.0) | 310,531 (100.0) | 6,063 (100.0) | 6,194 (100.0) | 20,309 (100.0) |

ARVC: arrhythmogenic right ventricular cardiomyopathy, CM: cardiomyopathy, DCM: dilated cardiomyopathy, HCM: hypertrophic cardiomyopathy, LVNC: left ventricular noncompaction, RCM: restrictive cardiomyopathy. Values are presented as N (column %). Patient counts are year-deduplicated and pooled across the 5-year study period. Column percentages are computed within each subtype using its column total as the denominator. Patients with missing or unspecified discharge-department codes (n = 15,763; 1.22%) were excluded from this analysis. Departments clinically appropriate for managing acute cardiomyopathy complications include cardiology, general internal medicine, intensive care unit, geriatrics, and general practice under China's hierarchical medical system. Cardiovascular-related departments managing cross-organ syndromes include pulmonary medicine, nephrology, and neurology, reflecting admissions for secondary organ complications of advanced cardiomyopathies.

**Figure S1**. Study flow diagram.

**Figure S2**. Temporal trends in age- and sex-standardized hospitalization rates for cardiomyopathies in China, 2018–2022, by subtype and sex. Each panel displays the overall (black solid line), male (blue dashed line), and female (red dashed line) standardized hospitalization rates per 1,000,000 population for the indicated cardiomyopathy subtype. Rates were standardized to the 2020 Chinese standard population derived from the Seventh National Population Census. Detailed numerical values, including APC, AAPC, and 95% confidence intervals, are reported in Table 1. CM, cardiomyopathy; DCM, dilated cardiomyopathy; HCM, hypertrophic cardiomyopathy; RCM, restrictive cardiomyopathy; ARVC, arrhythmogenic right ventricular cardiomyopathy; LVNC, left ventricular noncompaction.

**Figure S3**. Temporal trends in inpatient outcomes and resource utilization for cardiomyopathies in China, 2018–2022, by subtype. Panels display admission to intensive care unit (%), in-hospital mortality (%), median length of stay (days), and median hospitalization cost (CNY), respectively. The All-CM trend (black bold line) is shown alongside the five primary subtypes in each panel. All metrics are computed under the pre-specified patient-year analytic framework. Detailed numerical values are reported in Table 3. CM, cardiomyopathy; CNY, Chinese yuan; DCM, dilated cardiomyopathy; HCM, hypertrophic cardiomyopathy; LVNC, left ventricular noncompaction; RCM, restrictive cardiomyopathy; ARVC, arrhythmogenic right ventricular cardiomyopathy.
